# Recombinant GPI-Anchored TIMP-1 Stimulates Growth and Migration of Peritoneal Mesothelial Cells

**DOI:** 10.1371/journal.pone.0033963

**Published:** 2012-04-27

**Authors:** Roghieh Djafarzadeh, Matthias Sauter, Susan Notohamiprodjo, Elfriede Noessner, Pankaj Goyal, Wolfgang Siess, Markus Wörnle, Andrea Ribeiro, Susanne Himmelein, Thomas Sitter, Peter J. Nelson

**Affiliations:** 1 Arbeitsgruppe Klinische Biochemie, Medizinische Klinik und Poliklinik IV, Universität München, Munich, Germany; 2 Nephrologie, Medizinische Klinik und Poliklinik IV, Universität München, Munich, Germany; 3 Institute of Molecular Immunology, Helmholtz Zentrum München, Munich, Germany; 4 Institut für Prophylaxe und Epidemiologie der Kreislaufkrankheiten, Klinikum der LMU, München, Germany; University of Pittsburgh, United States of America

## Abstract

**Background:**

Mesothelial cells are critical in the pathogenesis of post-surgical intraabdominal adhesions as well as in the deterioration of the peritoneal membrane associated with long-term peritoneal dialysis. Mesothelial denudation is a pathophysiolocigally important finding in these processes. Matrix metalloproteinase (MMP) biology underlies aspects of mesothelial homeostasis as well as wound repair. The endogenous tissue inhibitors of metalloproteinases (TIMPs) moderate MMP activity.

**Methods and Finding:**

By modifying human TIMP-1 through the addition of a glycosylphosphatidylinositol (GPI) anchor, a recombinant protein was generated that efficiently focuses TIMP-1 on the cell surface. Treatment of primary mesothelial cells with TIMP-1-GPI facilitates their mobilization and migration leading to a dramatic increase in the rate of wound experimental closure. Mesothelial cells treated with TIMP-1-GPI showed a dose dependent increase in cell proliferation, reduced secretion of MMP-2, MMP-9, TNF-α and urokinase-type plasminogen activator (uPA), but increased tissue plasminogen activator (t-PA). Treatment resulted in reduced expression and processing of latent TGF-β1.

**Conclusions:**

TIMP-1-GPI stimulated rapid and efficient *in vitro* wound closure. The agent enhanced mesothelial cell proliferation and migration and was bioactive in the nanogram range. The application of TIMP-1-GPI may represent a new approach for limiting or repairing damaged mesothelium.

## Introduction

The peritoneum is a large serous membrane that covers intraabdominal organs (visceral peritoneum) and lines the peritoneal cavity (parietal peritoneum). The term peritoneal membrane is strongly associated with the application of peritoneal dialysis (PD). The peritoneal membrane consists of an innermost mesothelial cell monolayer, a basement membrane and the submesothelial stroma with extracellular matrix components, connective tissue cellular components and finally vascular and lymphatic structures. This membrane is used during PD as a semipermeable membrane that allows movement of urophanic substances and water in the abdominal cavity permiting the adjustment of electrolytes and acidbase homeostasis. Mesothelial injury by toxic, inflammatory (PD), mechanic or ischaemic (surgery) stimuli can lead to disturbance in the homeostatsis of the membrane. The identification of agents that could prevent or promote membrane repair is an important issue in mesothelial biology.

The MMPs are a large family of structurally related enzymes that collectively degrade extracellular matrix (ECM) [1]. The balance between MMPs and their endogenous inhibitors, the TIMPs, help to regulate ECM turnover during normal tissue homeostasis and pathogenesis. These proteins can also play key roles in moderating cell signaling through the cleavage of precursor proteins or proteolytic modification of cyokines or growth factors [2].

MMP/TIMP biology is important to peritoneal mesothelial cell homeostasis and repair [3]. Mesothelial cells can directly participate in the extracellular matrix turnover that follows serosal injury via elaboration of MMPs and TIMPs. The state of cellular differentiation appears to have an important influence on MMPs/TIMP expression such that epitheloid cells often display a more matrix-degradative phenotype (increased MMP and decreased TIMP) than their fibroblastoid counterparts [4].

GPI-anchored proteins are efficiently transferred from one cell to another through a process called cell painting or cell surface engineering [5], [6]. Modification of human TIMP-1 protein by the addition of a GPI anchor results in an agent that with enhance bioactivities which depend upon the cell system under study [6], [7], [8]. Recombinant TIMP-1-GPI fusion protein was shown to be readily incorporated into mesothelial cell surface membranes thus focusing the biologic actions of TIMP-1 directly onto the cell surface. We then evaluated the response of mesothelial cells to treatment with recombinant TIMP-1-GPI using a mechanical wound model and related *in vitro* assays. Our results demonstrate a strikingly accelerated wound closure rate following treatment of mesothelial cells with TIMP-1-GPI, as well as modulation of the fibrogenic milieu. These effects were linked in part to reduced TNF-α and TGF-β1 production by the mesothelial cells.

**Figure 1:**
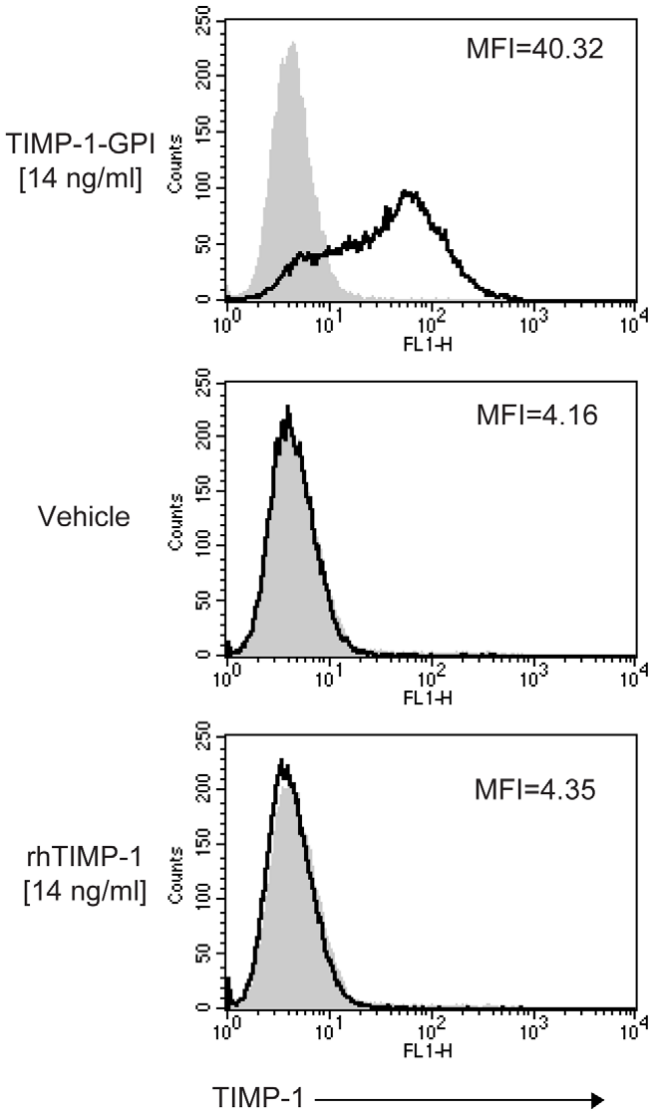
TIMP-1-GPI reincorporation into mesothel cells. To demonstrate the efficiency of reincorporation of GPI-anchored TIMP-1 protein into cell membranes, purified TIMP-1-GPI (14 ng/ml) or control rhTIMP-1 (14 ng/ml) was added to primary human mesothelial cells and TIMP-1 was then detected on the cell surface using an anti-human TIMP-1 monoclonal antibody and FACS analysis. The grey histograms represent isotype control stainings while the solid-line histograms represent TIMP-1 antibody signal.

## Materials and Methods

Medium M199 and newborn calf serum were obtained from Gibco BRL (Eggenstein, Germany), tissue culture plates were from Costar (Cambridge, MA, USA). Human serum was prepared from freshly collected blood of healthy donors and stored at –20°C. Fibronectin from human serum and trypsin were purchased from Boehringer (Ingelheim, Germany).

### Cell Culture Experiments

Primary human mesothelial cells were isolated from the omental tissue of consenting patients undergoing elective surgery as described in [9]. The studies were reviewed and approved by the University Ethics Committee. Cells were grown in fibronectincoated dishes in M199 medium supplemented with 25 mM Hepes (pH 7.3), 2 mM glutamine, 10% (vol/vol) human serum, 10% (vol/vol) newborn calf serum (heat-inactivated), penicillin (100 U/ml) and streptomycin (100 µg/ml) at 37°C under 5% CO_2_/95% air atmosphere. The medium was replaced every two to three days. Subcultures were obtained by trypsin/ethylenediaminetetraacetic acid (EDTA) treatment at a split ratio of 1∶3. Cells from omental tissue were human mesothelial cells as assessed by their uniform cobblestone appearance at confluence, by the absence of von Willebrand factor and the uniform positive staining for cytokeratins 8 and 18 and for vimentin. For the *in vitro* experiments, confluent cultures were used at the second or third passage, and cells were always given fresh media 48 hours before the experiment (M199 medium, supplemented with 2% (vol/vol) human serum and antibiotics). Confluent cells were demonstrated to be in a non-proliferative state under these conditions [10].

**Figure 2:**
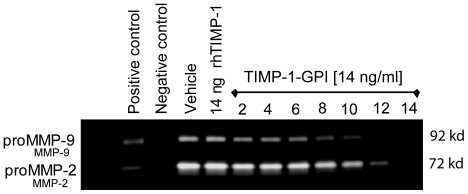
TIMP-1-GPI inhibits release of pro-MMP-2 and pro-MMP-9 from mesothel cells. TIMP-1-GPI inhibits the secretion of pro-MMP-2 and pro-MMP-9 from mesothelial cells. Gelatinase zymography was used to characterize the level of MMP-2 and MMP-9 proteins in mesothelial cell culture supernatants. The cells were treated with increasing amounts of TIMP-1-GPI (0−14 ng/ml), or control rhTIMP-1 (14 ng/ml), and after 48 h the serum-free culture supernatant was removed and analyzed on a gelatinase zymography gel [16].

**Figure 3:**
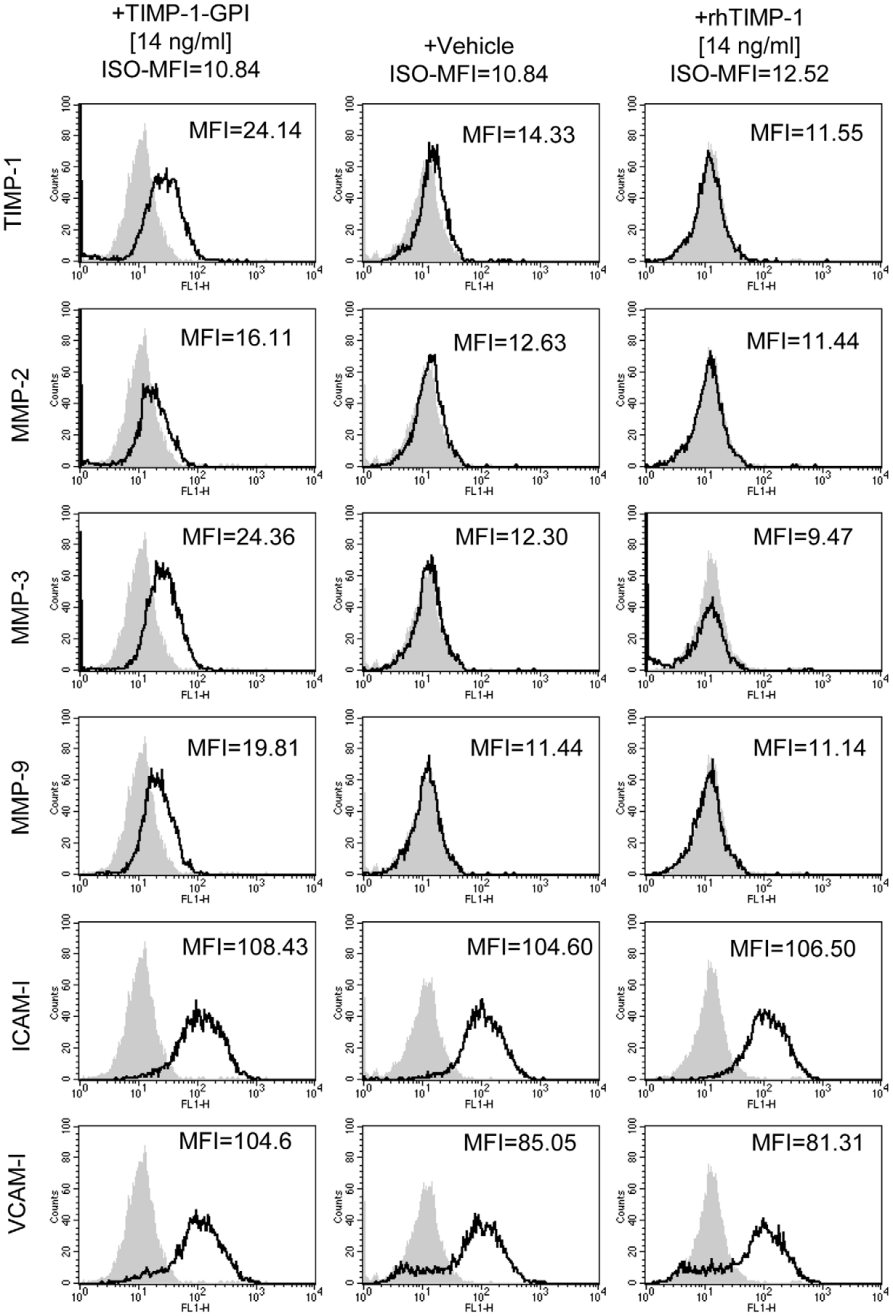
Effect of rhTIMP-1 and TIMP-1-GPI protein on the surface expression. Following incubation of mesothelial cells with 14 ng/ml of TIMP-1-GPI protein for 24 h, FACS analyses was performed using MMP-1, MMP-2, MMP-3, MMP-9, ICAM-1 and VCAM specific antibodies. The results show that treatment with TIMP-1-GPI, but not rhTIMP-1 lead to an increase in mean channel fluorescence intensity (MFI) for MMP-1, MMP-2, MMP-3 and MMP-9. VCAM and ICAM-1 are associated with the inflammatory status of the cells (they are involved in leukocyte recruitment). While VCAM was moderately increased, ICAM-1 levels remained unchanged with treatment.

### Fluorescence-activated Cell Sorting (FACS) Analysis

Cells were detached with 1.5 mM EDTA (Biochrom A, Berlin, Germany No. L2113) in 1×PBS and incubated for 60 min on ice with antibodies specific for human; TIMP-1 (IM32L), MMP-3 (IM36), MMP-2 (MS-806 PiABX) (Thermo Scientific Dreieich, Germany), MMP-9 a gift from Dr. Christian Ries (Chirurgische Klinik and Poliklinik, LMU), ICAM-1 (ICAM-I9GP89-11) a gift from Judith Johnson (Institute of Immunology, LMU) and VCAM-I (DAKO A/S, Glostrup, Denmark No. M7106) were described previously [6]. Cells were washed three times with 1×PBS, incubated with FTIC-conjugated anti-mouse mAB (DAKO A/S, Glostrup, Denmark No. F0313) for 45 min on ice, then washed three times with 1 x PBS and analyzed using a flow cytometer (FACSCalibur, Becton, Dickinson and Company, San Jose, CA, USA) and CellQuest software.

### Purification of TIMP-1-GPI Protein

The TIMP-1-GPI protein was produced and purified as previously described [6]. Briefly, human TIMP-1 was cloned from cDNA using hTIMP-1 specific primers, fused without a translation stop codon to the GPI-signal sequence cloned from LFA-3 [11], [12], subcloned into pEF-DHFR and stably introduced into DHFR deficient Chinese hamster ovary [13] cells and selected as described [14]. TIMP-1-GPI-fusion protein was subsequently purified from the CHO cells by Triton X-100 detergent extraction followed by column purification using DEAE, heparin sepharose and size exclusion as described [6].

### ELISAs and uPA Activity Assay

A human TIMP-1 specific ELISA kit was used to monitor levels of purified TIMP-1 in solution (BMS2018MST, Bender MedSystems GmbH, Vienna, Austria). ELISA for TNF-α and t-PA were performed on cell culture supernatants using commercial assay kits (Quantikin®, R&D Systems, Minneapolis, USA, No. 691228 and 762138) and t-PA ELISA from (TC Technoloclon, Wienna, Austria, No. TC12007). ELISA for PAI-1 was preformed on cell lysates using commercial assay kit (R&D Systems, Minneapolis, USA, No. DSE100).

UPA activity assay was performed on cell culture supernatants using commercial assay kits (SPECTROZYME® uPA, American Diagnostica GmbH, Pfungstadt Germany, No. 224L). Enzyme activity is determined by measuring the increase in absorbance of the free chromophore (pNA) generated per unit time at 405 nm. At excess substrate concentrations, the rate at which the absorbance increases due to the amount of chromophore released is linearly related to enzyme concentration. The Extinction Coefficient for SPECTROZYME uPA is 9650 M^−1^cm^−1^ and the concentrations were calculated through Beer-Lambert-Law.

#### TGF-β1 ELISA

Latent and active forms of TGF-β1 was measured using a commercial ELISA kit (DB Biosciences, San Jose, CA USA, No. 559119) used according the manufacture’s directions.

### Incorporation of TIMP-1-GPI into Cell Membranes

Primary human mesothelial cells (5−10×10^6^ cells/ml) were incubated with 14 ng/ml of purified hTIMP-1-GPI at 37°C/5% CO_2_ for 60 min. The cells were then washed one time with serum free medium and analyzed by FACS using human TIMP-1 specific monoclonal antibodies (see above).

### Proliferation

Primary human mesothelial cells (30×10^3^/100 µl medium) were cultured in 96-well micro-titer plates for 24 h under standard conditions to yield firmly attached and stably growing cells. After discarding supernatants, 50 µl of medium containing TIMP-1-GPI, buffer, or rhTIMP-1 was added to cells and incubated for 24 to 72 h. Then 50 µl of a 1 mg/ml solution of (3,5-Dimethylthiazol-2-yl] 2,5 diphenyl-tetrazolium bromide) MTT (SIGMA-ALDRICH, Taufkirchen, Germany No. M2128) was added. After 3-h incubation at 37°C, formazan crystals were dissolved by addition 100 µl isopropanol and 0.04 N HCl. Absorbance was then measured at 590 nm using GENios plus TECAN ELISA reader. For each experiment at least 6 wells were analyzed per experimental condition and time point.

#### Zymography

Primary human mesothelial cells were cultured in 24 wells plate (5×10^4^ cells/well). The medium was exchanged for 24 h with serum-free medium containing either rhTIMP-1, or other controls and increasing amounts of TIMP-1-GPI and incubated for 24 h, 48 h and 72 h. Cell supernatants were analyzed by gelatin zymography using 10% SDS-polyacrylamide gels (Invitrogen, Groningen, Netherlands, No. EC61755BOX) as described [6]. Recombinant MMP-9 enzyme (Amersham Biosciences, Uppsala, Sweden, No. RPN2634) was used as positive control.

**Figure 4:**
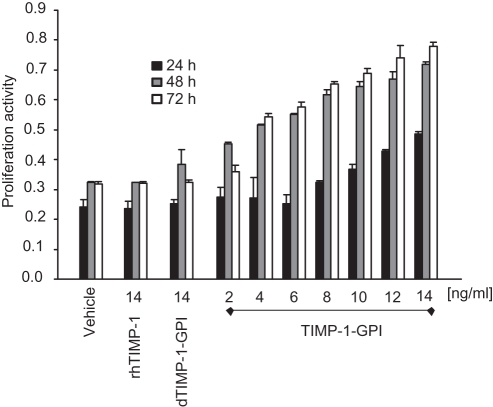
Effect of rhTIMP-1 and TIMP-1-GPI protein on the proliferation of human mesothel cells. The effect of increasing levels of TIMP-1-GPI or rhTIMP-1 control protein on the proliferation of primary human mesothelial cells was measured using an MTT assay [49]. MTT was added after 24, 48 or 72 h as indicated. TIMP-1-GPI treatment was found to enhance proliferation of the mesothelial cells while no effect was seen with rhTIMP-1 or dTIMP-GPI.

**Figure 5:**
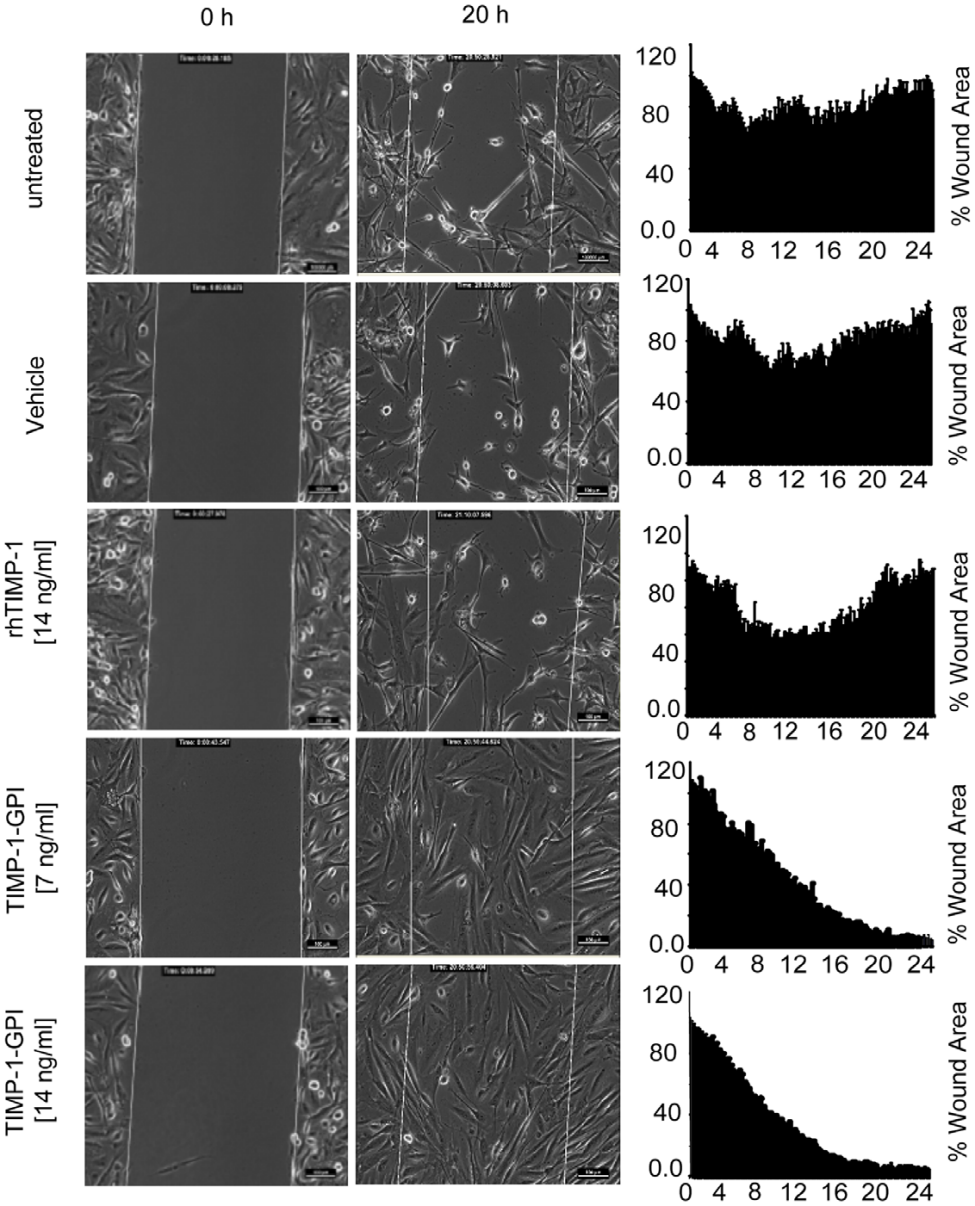
TIMP-1-GPI promotes wound healing *in vitro.* A monolayer of human primary mesothelial cells was cultured with an experimental wound model insert provided by Ibidi GmbH (Munich, Germany). Removal of the insert yielded a uniform “wound” to the monolayer. (**A**) Treatment with TIMP-1-GPI but not the control proteins, showed a dose dependent increase in wound closure. (**B**) The closing areas were calculated using imaging software NIS-Elements. (Video S1, S2, S3, S4, S5).

#### Wound healing assay

Uniform wounds were introduced into the cell culture system by using the ibidi Culture-Insert (ibidi GmbH Munich, Germany, No. 80209). This approach provides two cell culture reservoirs with a separation wall of 500 µm thick. The culture-Inserts were placed in the individual wells of a six well plate. In each reservoir, 3000 mesothelial cells were cultured with an end volume of 100 µl. The silicon inserts were removed after the cells had undergone adherence. The gaps created (or wounds) were washed with serum-free medium and each well was filled with 2 ml sample (medium, vehicle, 14 ng/ml rhTIMP-1 or TIMP-1-GPI).

Wound closure was then monitored by tracing the wound area using recording. Images were acquired every 10 min using a Meta Morph controlled Nikon camera (Fluorescence Inverted microscope (Nikon 2000 E, Nikon GmbH Duesseldorf, Germany) at 37°C by CO_2_ supply for 24 h. The microscope system was pre-heated for several hours before starting the experiment. The microscope function was controlled by NIS elements software. Pictures were taken every 7 min for 24 h. Movie was edited with QuickTime Pro and iMovie software from Apple Inc. The closing areas were calculated using imaging software NIS-Elements (Nikon GmbH Duesseldorf, Germany). The software was used to determine changes in the wound surface over time. The data was automatically incorporated into Excel program for further analysis.

**Figure 6:**
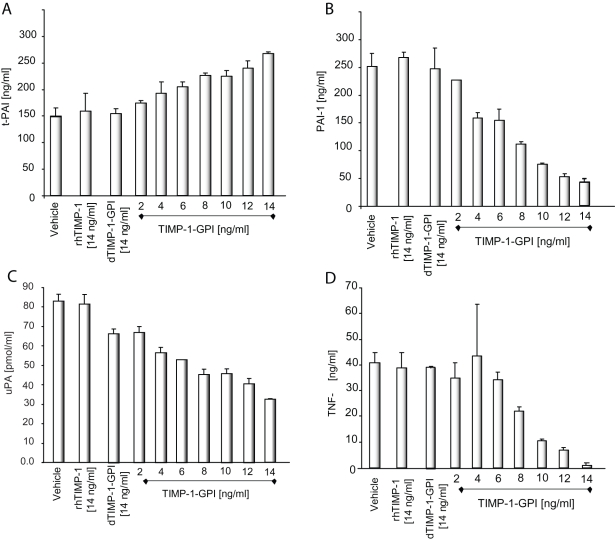
The effect of TIMP-1-GPI on t-PA, PAI-1, TNF-α and uPA protein secretion on human mesothelial cells. A quantitative assessment of protein expression was analyzed using specific ELISA and activity assays after 12 h TIMP-1-GPI treatment. (**A**) t-PA release was increased in dose dependent manner of TIMP-1-GPI whereas the secretion of PAI-1 (**B**) decreased under the same conditions. (**C**) The release of uPA was decreased in human mesothelial cells when the cells after 12 h treatment with TIMP-1-GPI. (**D**) TNF-α protein was also reduced by treatment with TIMP-1-GPI. Denatured dTIMP-GPI had no effect.

**Figure 7:**
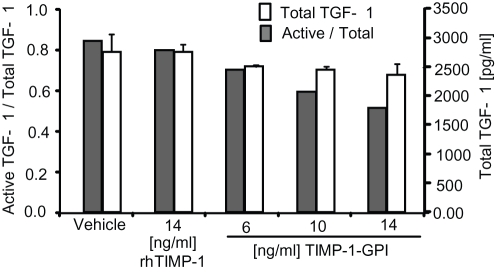
TIMP-1-GPI treatment reduces activation of TGF-β1. Primary human mesothelial cells were treated with increasing amounts of TIMP-1-GPI, the culture supernatant was harvested at 24 h and the total and active levels of TGF-β1 was determined.

## Results

### Cell Surface Engineering of Primary Human Mesothelial Cells by the Addition of Recombinant TIMP-1-GPI

GPI-anchored TIMP-1 protein was generated and isolated as previously described [6], [7], [8]. The efficiency of incorporation of recombinant GPI-anchored TIMP-1 protein into the surface membranes of primary human mesothelial cells was determined after incubation of the cells with 14 ng/ml of purified TIMP-1-GPI, recombinant human (rh)TIMP-1 control protein or vehicle for one hr at 37°C. Surface associated TIMP-1 protein was detected using fluorescence-activated cell sorting (FACS) using an anti-human TIMP-1 monoclonal antibody (**Figure 1**). Addition of vehicle or control rhTIMP-1 protein did not lead to a change in the FACS shift. Treatment with GPI-anchored TIMP-1 resulted in a strong surface signal for human TIMP-1 protein.

### Cell Surface Associated TIMP-1-GPI Protein Lead to a Block in the Release of proMMP-2 and proMMP-9 from Mesothelial Cells

Human primary mesothelial cells constitutively secrete both proMMP-2 and proMMP-9 proteins as seen by gelatinase zymography (**Figure 2**). These proteins have been previously linked to mesothelial biology [15]. The effect of increasing surface TIMP-1 protein levels on the secretion of MMP-2 and MMP-9 proteins was evaluated [6], [16]. While treatment with vehicle or rhTIMP-1 protein (14 ng/ml) had no effect on MMP-2 or MMP-9 secretion, by contrast, starting at 8 ng/ml, addition of TIMP-1-GPI lead to a concentration-dependent decrease in the release of both MMP-2 and MMP-9 into the growth media.

### Cell Surface Engineering with TIMP-1-GPI is Associated with an Increase in Cell Surface Associated MMPs

TIMP-1 is known to bind most active forms of MMPs. Based on the results of the gelatinase experiments, it was suggested that TIMP-1-GPI could potentially sequester the normally secreted MMPs on the surface of the TIMP-1-GPI “engineered” cells. Following incubation of mesothelial cells with 14 ng/ml of TIMP-1-GPI protein for 24 h, FACS analyses using MMP-1, MMP-2, MMP-3 and MMP-9, showed an increase in mean channel fluorescence intensity (MFI) for MMP-1, MMP-2, MMP-3, and MMP-9 on the TIMP-1-GPI treated cells, but not on the rhTIMP-1 control cells. ICAM-1 and VCAM-1 expression is associated with leukocyte recruitment to damaged mesothelium. Specific antibodies directed against these proteins were used to evaluate the effect of treatment on their expression. While ICAM-1 remained unchanged, a moderate increase in VCAM-1 was seen (**Figure 3** **).**

### Treatment of Primary Human Mesothelial Cells with GPI-anchored TIMP-1 Lead to a dose Dependent Increase in their Proliferation

We have previously shown that treatment with TIMP-1-GPI can either enhance or suppress cell proliferation depending on the specific cell type studied [6], [7], [8]. To assess the effect of TIMP-1 surface engineering on the proliferation of primary mesothelial cells *in vitro*, MTT assays were performed. The exogenously added TIMP-1-GPI protein was found to elicit a dose-dependent increase in the proliferation of primary human mesothelial cells (**Figure 4**).

### Treatment with TIMP-1-GPI Stimulates Rapid Mesothelial wound Closure in an *In Vitro* Model of Mesothelial wound Healing

To help characterize the effect of TIMP-1 surface engineering on directional cell migration as associated with wound repair, an *in vitro* wound-healing model was applied and the effect of TIMP-1-GPI treatment evaluated. The approach involves creating a “wound” on a monolayer of primary human mesothelial cells using a commercial apparatus (ibidi, Munich, Germany), treatment of the cells with either recombinant TIMP-1-GPI protein or control protein, and then capturing video images from the initiation of the experiment through to wound closure. The combined migration rate and proliferation effect was then quantified using imaging software (NIS-Elements, see Materials and Methods).

While treatment of the “wounded” primary mesothelial cell monolayer with growth medium, vehicle or rhTIMP-1 protein alone lead to only limited wound closure by 24 h, application of TIMP-1-GPI at 7 ng/ml or 14 ng/ml lead to a rapid wound closure by 20 or 16 h respectively (**Figure 5**) (Video S1, S2, S3, S4, S5).

### TIMP-1-GPI Treatment Lead to a Reduction in uPA, PAI-1 and TNF-α Secretion/Activity Levels, but Increased t-PA by Human Mesothelial Cells

Tissue plasminogen activator (t-PA) and the urokinase plasminogen activator (uPA) are fibrinolytic agents while plasminogen activator inhibitor-1 (PAI-1) inhibits their activity. Alterations in these proteins have been linked to PD and general mesothelial damage [17], [18], [19], [20]. While mesothelial cell surface engineering with TIMP-1-GPI lead to a dose dependent increase in t-PA secretion, a decrease in uPA and PAI-1 secretion was seen by 12 h (**Figure 6** **A-C**). The observation that tPA is enhanced, while uPA and PAI1 are reduced can be explained in part by the effect of TIMP-1-GPI treatment on the steady state mRNA expression of these genes **(Text S1)**. While TIMP-1-GPI lead to an increase in steady state mRNA levels of t-PA, a reduction in PAI-1 mRNA was seen (**Figure S1**).

TNF-α is a general activator of inflammatory processes and can also promote apoptosis of mesothelial cells [21]. [22]. Treatment of the human mesothelial cells with TIMP-1-GPI lead to a dose dependent reduction in TNF-α release and reduced steady state mRNA expression (**Figure 6** **D, Text S1 and Figure S1**).

### Treatment Leads to Reduction in the Processing and Secretion of TGF-β1

Mesothelial proliferation and migration can be inhibited by TGF-β1 [23]. TGF-β1 is also strongly associated with wound healing in general [24], [25]. ELISA was used to assess the secretion of total vs. active forms of TGF-β1 by primary mesothelial cells with and without treatment with TIMP-1-GPI or control protein. While total TGF-β1 was moderately reduced by treatment with TIMP-1-GPI (**Figure 7**), treatment led to reduction in active TGF-β1 suggesting an effect on proteolytic processing of the latent TGF-β1 complex (**Figure 7**).

**Figure 8:**
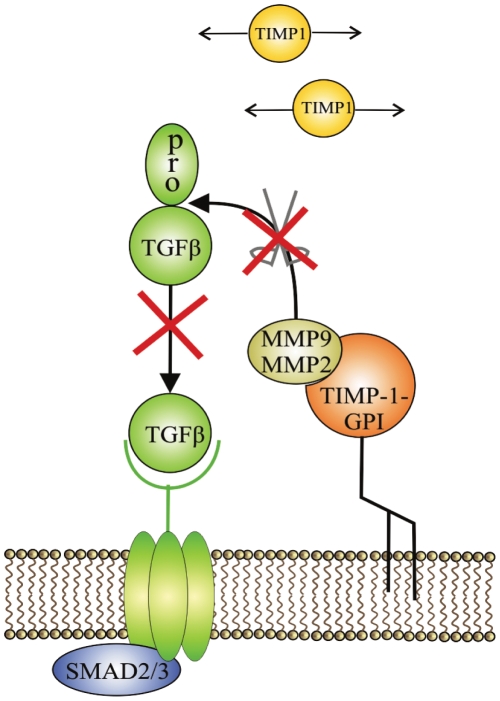
TIMP-1-GPI impacts TGF-β1 expression and processing. TGF-β1 can inhibit mesothelial cell migration and proliferation. MMP-9 and MMP-2 can proteolytically activate latent TGF-β1. Cell surface engineering with TIMP-1-GPI leads to a reduction in the expression and processing of latent TGF-β1 to its active form.

## Discussion

Wound healing involves the orchestration of a series of cellular and biochemical events many of which are linked to aspects of MMP/TIMP biology [26], [27], [28]. We evaluated the effect of a novel TIMP based reagent on processes associated with mesothelial repair. Recombinant TIMP-1-GPI was shown to be efficiently incorporated into cell membranes leading to a shift in the association of TIMP functional domains from the pericellular environment to the cell surface (**Figure 8**). This treatment led to moderation in the expression of cytokines and growth factors by mesothelial cells. TIMP-1-GPI also reduced MMP-2, MMP-9, TGF-β1 and TNF-α secretion, and altered the balance between uPA, t-PA and PAI-1 expression. The reduced gelatinase production seen may result in part from the sequestering and blockade of MMPs directly on the surface of treated cells. Treatment also led to enhanced mesothelial cell proliferation and dramatically enhanced migration/repair in an *in vitro* wound healing assay (**Figure 7** **and** **8**).

Mesothelial cells represent the largest population of resident cells in the peritoneal cavity. They provide a non-adhesive and protective layer against foreign particles and injury to the peritoneum [29]. Mesothelial cells play a central role in the process and function of peritoneal dialysis. Any morphologic changes of the mesothelium are likely to have an impact on PD [30], [31]. During normal mesothelial repair, cells in the peritoneal fluid implant in depopulated peritoneal areas. Mesothelial cells can also migrate from the connective tissue to the peritoneal surface and replicating cells from the borders of denuded zones become mobilized and migrate to facilitate repair [32]. Thus, mobilization and proliferation of these cells is central to efficient wound healing.

MMPs play diverse roles in wound healing [33]. They degrade the ECM. A balance between this activity and ECM production underlies optimal wound healing. MMP biology is also associated with the regulation of cell cycle and cellular proliferation [34], [35]. MMP activity is controlled by four endogenous TIMP proteins (TIMP-1, TIMP-2, TIMP-3 and TIMP-4), which are produced by most cells, and inhibit MMPs in a 1∶1 ratio.

TIMPs can have diverse biologic effects that depend on the context of their expression [36]. We have previously shown that engineering TIMP-1 by the addition of a GPI anchor results in a recombinant protein that has enhanced and novel bioactivities depending on the cell type studied and activation status [6], [7], [8].

TIMP-1-GPI treatment of mesothelial cells in the context of a mechanical *in vivo* wound healing model demonstrated early and efficient wound closure. This effect was associated with enhanced proliferation, mobilization and migration of the cells.

Mesothelial cells express adhesion molecules, including intercellular adhesion molecule-1 (ICAM-1) and vascular cell adhesion molecule-1 (VCAM-1) [37], which help facilitate leukocyte movement into the peritoneal cavity [38]. TIMP-1-GPI did not influence ICAM-1 expression on the surface of treated cells, but did moderately increase VCAM-1 expression.

In addition to mesothelial denudation, a hypercoagulatory state in the peritoneal cavity can also contribute to the thickening of the peritoneal membrane in chronic inflammation [39] and during adhesion formation following surgical procedures [40]–[41]. Mesothelial cells produce components of a plasma independent fibrinolytic system: t-PA and uPA are fibrinolytic agents while PAI-1 inhibits their fibrinolytic potential. Disturbances in the balance of the fibrinolytic agents or their inhibitor have been demonstrated following surgical trauma episodes or PD associated peritonitis, or after stimulation of cultured mesothelial cells with proinflammatory agents [19], [20], [42].

Treatment with TIMP-1-GPI led to a dose dependent decrease in PAI-1 and an increase in t-PA secretion, as well as a decrease in uPA production. t-PA plays a pivotal role in the fibrinolytic system by converting the proenzyme plasminogen in the active enzyme plasmin, a potent broad-spectrum protease that cleaves fibrin. Moreover plasmin is able to degrade several components of extracellular matrices by activating latent procollagenases and metalloproteases [43], [44]. Mesothelial uPA production is of minor importance for the intraperitoneal fibrinolytic system, as t-PA activity exceeds it by far [13]. Treatment with TIMP-1-GPI shifted this balance to fibrinolysis that occurs in conjunction with facilitated wound repair.

Human mesothelial cells undergo apoptosis during peritonitis which may be related to the resolution of peritoneal inflammation [45]. Elevated levels of TNF-α are found in the peritoneal fluid from CAPD patients undergoing episodes of peritonitis [21]. TNF-α directs mesothelial cells to undergo apoptosis via the Fas/Fas ligand pathway [22]. Mesothelial cells can be a source of intraperitoneal TNF-α, especially in response to noxious stimulants [46]. Treatment with TIMP-1-GPI lead to reduced mesothelial TNF-α release. In addition, treatment also moderately reduced the expression of FAS receptor expression on the surface of TIMP-1-GPI-treated cells. TIMP-1-GPI-treated cells showed approximately 20% less FAS receptor expression (by FACS) than that seen in controls (data not shown).

The role of MMP/TIMP biology in cellular proliferation and migration is complicated and appears to depend upon the type and activation state of the cells involved [6], [7], [8]. MMPs can moderate autocrine and paracrine factors linked to these processes. Many factors such as TGF-β1 and TNF-α are synthesized as precursor proteins that require proteolytic processing to gain functional activity [47], [48]. TGF-β1 can effectively block the migration and proliferation of mesothelial cells [24], [25]. MMP-2 and MMP-9 activate TGF-β through proteolytic degradation of the latent TGF-β1 complex [47]. These MMPs are sequestered at the cell surface by TIMP-1-GPI treatment. This is associated with a reduction in the processing of TGF-β1 to its active form, and in parallel, a reduction in TNF-α secretion and steady state mRNA.

Exogenously applied TIMP-1-GPI fusion protein is incorporated into mesothelial surface membranes leading to a direct focusing the biologic actions of TIMP-1 onto the cell surface. This surface engineering has robust effects on mesothelial biology. Treatment reduced MMP release and leads to accelerated wound closure through the activation of proliferation and migration of mesothelial cells and moderated the fibrogenic environment. Treatment with TIMP-1-GPI may show efficacy in the repair of damaged mesothelium.

## Supporting Information

Text S1 **RT-PCR analyses.**
(DOC)

Figure S1 **TIMP-1-GPI treatment moderates the steady state TNF-α, PAI-1 and t-PA gene expression.** Primary human mesothelial cells were left untreated, or were treated with 7 ng/ml TIMP-1-GPI, 14 ng/ml TIMP-1-GPI, 14 ng/ml of heat treated TIMP-1-GPI or 14 ng/ml of rhTIMP-1. After 48 hrs RNA was isolated and subjected to analysis using TaqMan RT-PCR. The genes analyzed were **(A)** t-PA, **(B)** TNF-α and **(C)** PAI-1.
(EPS)

Video S1 **Medium control.**
(AVI)

Video S2 **Vehicle control.**
(AVI)

Video S3 **Control rTIMP-1 14 ng/ml.**
(AVI)

Video S4 **TIMP-1-GPI 7 ng/ml.**
(AVI)

Video S5 **TIMP-1-GPI 14 ng/ml.**
(AVI)
