# Supplementary material for: Recombinant GPI-Anchored TIMP-1 Stimulates Growth and Migration of Peritoneal Mesothelial Cells
Source: PLoS One. 2012 Apr 27;7(4):e33963. doi: 10.1371/journal.pone.0033963 (PMC3338742; doi:10.1371/journal.pone.0033963)
Supplement: Text S1 — RT-PCR analyses. (DOC) [file pone.0033963.s001.doc]

**Text S1**

### **RT-PCR analyses**

Total mesothelial RNA was isolated for standardized quantitative RT-PCR by using RNeasy MiniKit 50 (Quiagen, Hilden, Germany, No. 74104). In brief, 2 *µ*g of total RNA from cultured primary human mesothelial cells underwent random primed reverse transcription using a modified MMLV reverse transcriptase (Superscript II, Life Technologies, Karlsruhe, Germany) for 1 h at 40°C. Real time RT-PCR was performed on a TaqMan 7000 Sequence Detection System (Applied Biosystems, Warrington, UK) using heat activated TaqDNA polymerase (Amplitaq Gold, PE Biosystems, Weiterstadt, Germany). After 2 min at 50°C and 10 min at 95°C the samples were cycled 45 times at 95°C for 15 sec and 60°C for 60 sec. PCR products were labeled by internal fluorescence probes (TNF-α, PAI-1 and t-PA and β-Actin) mRNA expression for each signal was calculated following the ΔCt procedure . Commercially TaqMan reagents were obtained from Applied Biosystems, Warrington, UK.
